# Use of the life table to compare mortality in ethnic groups in Amsterdam, the Netherlands

**DOI:** 10.1186/s12889-015-2170-y

**Published:** 2015-08-27

**Authors:** Daan G. Uitenbroek

**Affiliations:** Public Health Service Amsterdam, Department of Epidemiology and HealthPromotion, Nieuwe Achtergracht 100, P O Box 2200, Amsterdam, 1000 CE Netherlands

## Abstract

**Background:**

The life table is a valid and frequently used instrument to compare the mortality of migrant groups. Most analyses are limited to an overview and give only life expectancy; however, further analysis of the life table can give more insight into differences in patterns of mortality between groups.

**Methods:**

A thorough life table analysis was applied to the mortality data of seven ethnic groups by age and gender.

**Results:**

Life expectancy is systematically higher in migrants compared with the Dutch citizens of Amsterdam. However, between birth and the age of 40 the probability of death is higher among non-western migrants compared with citizens of western origin. The number of deaths is small among the young. This results in very small differences in survival between the groups; from birth up to the age of 40 the survival rate is 98.7 % for citizens of western origin and 98.3 % for citizens of non-western origin. In all seven ethnic groups over 90.7 % of babies, male and female, survive up to the age of 60. In all female groups the survival is better than in male groups. Males and females aged 0 to 40 from Antillean origin are the only exception.

**Conclusion:**

Life expectancy is generally higher in non-western than in western groups. Differences in survival between ethnic groups are small up to middle age.

## Background

Life expectancy is a basic measure to summarise the mortality of different ethnic groups by origin [1]. Life expectancy has the advantage of being an intuitively easy to understand measure, also to non-statisticians. Finding differences in life expectancy between groups could be the starting point of an analysis of causes of death. This can be done by collecting additional information, for example on causes of death or associated morbidity. However, the starting point for further study could also be the already available life table on which the life expectancy calculation is based.

Differences in life expectancy between origin groups in Amsterdam were previously reported [2]. Life expectancy was generally higher among citizens of non-Dutch origin compared with the Dutch. This healthy migrant effect is an international phenomenon. It relates to the fact that migrants often have a higher life expectancy although they are mostly social-economically disadvantaged compared with the indigenous population. Possible causes are a healthier lifestyle, the “Mediterranean effect”, and genetic predisposition. Methodological problems mentioned are problems such as the under-registration of deaths abroad, and people remigrating without deregistering, as a result of which the population size of migrant groups are estimated too high [3–5]. Emigration of relatively healthy Dutch people to suburban towns is considered to be an important possible cause of the comparatively low life expectancy of ethnically Dutch people living in the city of Amsterdam [2]. An analysis of age-specific death rates shows that in younger age groups immigrants have a considerably higher mortality, although the numbers of deaths are relatively small, while in older age groups the Dutch have a slightly higher mortality, but now with much higher numbers of deaths [2, 6]. This supports the hypothesis that lifestyle, which is related to chronic diseases in older age, causes the healthy migrant effect. However, although this was not investigated, it suggests also that although the Dutch have a lower life expectancy, it might well be that they have a good probability to reach an older age. That is because their mortality at a young age is comparatively low.

To investigate this and other patterns of mortality between ethnic groups, this paper studies differences in the life tables for the different origin groups using various methods.

## Methods

Age-specific numbers of deaths and population size for the years 2010–2014 were provided by the Office for Research and Statistics of the city of Amsterdam. Number of deaths and population data relate exclusively to people legally residing in Amsterdam. In the context of combating tax and benefit fraud there is a major effort to keep registration data complete. Registrations from the municipal personal records database, the city of Amsterdam, the tax authorities, housing associations and utility companies are part of this effort. Returned, non-answered and inappropriately answered mail is followed-up by house visits, visits to family members and neighbours. The municipal personal records database, on which the mortality and population of this study is based, is therefore generally considered to be complete and up to date. However, there will be differences between origin groups in terms of this completeness, which may influence the results of this study. The number of deaths for the five years ranged from 95 for Antillean females to 10,854 for Dutch females. Denominators ranged from 29,647.5 person years for Antillean males to 1,006,538 person years for Dutch females.

Respondents are classified of foreign origin if the individual or one or both of the parents are foreign born. Precedence is given first to the place of birth of the person, but if one of the parents is foreign-born, precedence is given to the parent’s origin. Western populations basically relate to people living in the industrialised countries, such as North America, Europe and Japan, while non-Western relates to the non-industrialised countries, for example all of Africa and most of Asia [7].

Chiang’s [8] methods are used to construct the life tables and to calculate the variance for the survival probabilities and the life expectancy. The variance of Chiang’s method is used to calculate the x ± 1.96*sqrt(var(x)) confidence intervals for both mortality probabilities and the life expectancy. Standard errors and confidence intervals were mostly very small and are therefore not included in all tables.

Population and mortality data used in this paper and life table calculations are available at: http://www.quantitativeskills.com/ggd/LEtab1.xlsx and http://www.quantitativeskills.com/ggd/LEtab2.xlsx. Please note that the spreadsheets are computing intensive and might take long to load and slow to handle in excell.

## Results

Table 1 shows a number of columns of a life table for citizens living in Amsterdam of western origin, and the same columns for citizens of non-western origin. This is not a full life table; it only shows the columns relevant for the discussion in this paper. Column qi in the first row of Table 1 shows the probability of dying between birth and the first birthday. This probability is 0.38 % for Amsterdam citizens of Western origin, and 0.51 % for citizens of non-western origin (t = 2,12; p < 0.05). Thus, the probability of dying between birth and the first birthday is 1.34 times (=0.51/0.38) larger for citizens of non-western origin compared with citizens of western origin. However, mortality is small for both groups, less than one percent of the total population aged less than one. Even smaller than the chance to die between birth and the first birthday is the chance to die between the first and the 20th birthday, 0.26 % for Western and 0.27 % for non-western citizens (t = 0.02; p > 0.05). Between the 20th and 40th birthday the probability to die is 1.37 (=0.0093/0.0068) times higher for non-western compared with western citizens (t = 3.53; p < 0.05). Column li shows the number of individuals in a hypothetical cohort which is exposed to the mortality probabilities in column qi. The data in this column can be used to calculate survival probabilities between ages. This way, the probability to survive from birth to age 40 is 98.7 % (=98680/100000) for a citizen of Amsterdam of western origin, and 98.3 % for a citizen of non-western origin. The differences in life expectancy are highly significant for all rows (all t > 20).

**Table 1:** Life table entries for western and non-western groups living in Amsterdam

Age	Western {p = 2588752; d = 23168}			Non-western {p = 1387498; d = 3637}		
I	qi	Li	ei	qi	li	ei
0	0.0038	100000	79.9	0.0051	100000	81.7
1	0.0026	99618	79.2	0.0027	99490	81.2
20	0.0068	99354	60.4	0.0093	99225	62.3
40	0.0334	98680	40.8	0.0289	98300	42.8
55	0.0780	95315	27.1	0.0561	95390	29.0
65	0.0693	87856	18.9	0.0644	89770	20.5
70	0.1104	81768	15.1	0.0926	83989	16.7
75	0.1802	72738	11.7	0.1734	76210	13.2
80	1.0000	59632	8.7	1.0000	62995	10.4

Age: Age at the beginning of interval i

qi: Probability at the “i-th” birthday to die before reaching age “i + 1”

li: Size of a hypothetical birth cohort at birthday “i”

ei: Life expectancy at birthday “i”

{p = life years; d = number death}

After the age of 40 the differences in mortality of citizens of western and non-western origin are reversed. As previous studies have shown, chronic diseases become important and chronic diseases are, in general terms, more common among populations of western origin (Bos et al., 2004). The relative difference in mortality between western and non-western citizens is not as high as in younger age groups, but as the percentages are higher, it concerns more people dying. In terms of survival, at their 40th birthday citizens of western origin have an 82.9 % (=81768/98680x100) chance of reaching age 70, citizens of non-western origin have an 85.4 % chance of becoming 70.

To study to what extent the findings are explained by averaging the differences between groups, Table 2 presents the data again for three age bands, but with more origin groups in the rows and also considering differences between males and females. The qi column of Table 2 shows that the probability of a baby boy born in Amsterdam dying before the age of 40 is 1.81 %. There are large differences between the origin groups: baby boys from Surinam and other non-western origin have a higher probability to die before the age of 40 than babies from Dutch and western origin. Mortality is lowest for citizens of Antillean origin. Given the low number of cases, chance fluctuation will have played a role for this group, but also, given previously high baby and youth mortality among Antillean males, this group has been extensively focused on in care and prevention. In the middle age group, between 40 and 60, there is no systematic difference between western and non-western groups. The Dutch have a higher (7.84 %) and the other western groups a lower (7.26 %) mortality probability compared with the city average of 7.35 %; of the non-western groups those of Surinam and Antillean origin have a higher and the Moroccan, Turkish and other non-western groups have a lower mortality probability compared with the city average. Similarly, in the age group between 60 and 80, the differences do not seem systematic between western and non-western origin groups. However, using t-tests (not shown in the table), in Table 2 none of the mortality probabilities for the groups differs significantly from the overall city probability. The li column shows that survival for males from birth up to the age of 60 ranges from 89.0 % for males from Surinam origin to 93.7 % for males of Moroccan origin. The city average is 91.2 %. In the last columns of the table the life expectancy with confidence interval is shown. The life expectancy for males in Amsterdam is 78.2 years. In all age groups males from Dutch decent have have a lowt life expectancy, which is statistically significant for age forty and sixty. Males of Moroccan, Turkish and other non-western decent have a systematically and statistically significantly higher life expectancy.

**Table 2:** Life table entries for a number of different origin groups living in Amsterdam, males. (Between brackets WHO notation) {p = life years, d = number death} **: + = life expectancy significantly better than city average (“all groups”), − = significantly wears

	qi	95 % CI		li	ei	95 % CI		**
Age 0 to 40 (40q0)								
All groups {p = 1091424, d = 547}	0.0181	0.0165	0,0198	100000	78.0	77.9	78.2	
Dutch {p = 477855, d = 233}	0.0167	0.0143	0,0192	100000	77.8	77.5	78.0	
Other western {p = 168980, d = 66}	0.0136	0.0099	0,0173	100000	78.6	78.2	79.1	+
Moroccan {p = 125871, d = 64}	0.0208	0.0159	0,0257	100000	82.3	81.5	83.1	+
Antillean {p = 18389, d = 4}	0.0069	0.0000	0,0153	100000	79.0	77.1	80.9	
Surinam {p = 90775, d = 55}	0.0234	0.0172	0,0296	100000	77.2	76.5	78.0	-
Turkish {p = 72798, d = 35}	0.0188	0.0126	0,0251	100000	80.0	78.4	81.5	+
Other non-western {p = 136758, d = 90}	0.0240	0.0187	0,0293	100000	80.1	79.1	81.1	+
Age 40 to 60 (20q40)								
All groups {p = 566684, d = 1961}	0.0735	0.0707	0,0764	98195	39.1	39.0	39.3	
Dutch {p = 287684, d = 1098}	0.0784	0.0742	0,0826	98338	38.8	38.6	39.0	-
Other Western {p = 86604, d = 280}	0.0726	0.0655	0,0797	98647	39.4	39.0	39.8	
Moroccan {p = 40924, d = 83}	0.0440	0.0357	0,0524	97933	43.6	42.8	44.4	+
Antillean {p = 8332, d = 37}	0.0892	0.0630	0,1154	99313	39.3	37.4	41.2	
Surinam {p = 48597, d = 219}	0.0914	0.0805	0,1023	97673	38.5	37.9	39.2	-
Turkish {p = 27728, d = 78}	0.0682	0.0564	0,0800	98126	41.1	39.6	42.6	+
Other non-western {p = 66817, d = 166}	0.0535	0.0463	0,0607	97619	41.7	40.7	42.6	+
Age 60 to 80 (20q60)								
All groups {p = 260886, d = 5559}	0.4879	0.4804	0,4953	91145	21.4	21.2	21.5	
Dutch {p = 164015, d = 3646}	0.4986	0.4892	0,5080	90818	21.1	21.0	21.3	
Other western {p = 39050, d = 824}	0.4871	0.4679	0,5063	91648	21.6	21.2	22.1	
Moroccan {p = 16685, d = 266}	0.3633	0.3352	0,3914	93682	25.2	24.4	25.9	+
Antillean {p = 2814, d = 53}	0.4850	0.4145	0,5556	90713	22.3	20.3	24.2	
Surinam {p = 17927, d = 404}	0.5352	0.5067	0,5637	89007	21.3	20.7	22.0	-
Turkish {p = 7510, d = 162}	0.5300	0.4861	0,5739	91577	23.3	21.7	24.8	+
Other non-western {p = 12886, d = 204}	0.4202	0.3883	0,4522	92491	23.4	22.5	24.4	+

Table 3 shows the data for females. The pattern for baby girls is similar to the pattern for baby boys. It can be seen that the probability of a baby girl born in Amsterdam dying before the age of 40 is 1.12 %. There are large differences between the origin groups: baby girls of Antillean (1.42 %) and other non-western origin (1.49 %) have a higher probability to die before the age of 40 than babies from other western (0.65 %), Turkish (0.97 %) and Moroccan origin (0.92 %). Both in the age group 40 to 60 and in the age group 60 to 80, females from Dutch origin have a high mortality probability, and females from Turkish and Moroccan origin have a low mortality probability. As for males, in Table 3 none of the mortality probabilities for the groups differs significantly from the overall city probability. The li column shows that survival for females from birth up to the age of 60 ranges from 93.2 % for females of Dutch origin to 96.5 % for females of Turkish origin. The city average is 94.1 %. The life expectancy for females in Amsterdam is 82.3 years. In life expectancy terms, females from Dutch origin perform rather poor; females from all other origins do better, often statistically significantly so.

**Table 3:** Life table entries for a number of different origin groups living in Amsterdam, females

	qi	95 % CI		li	ei	95 % CI		
Age 0 to 40 (40q0)								
All groups {p = 1138531, d = 339}	0.0112	0.0100	0,0125	100000	82.1	82.0	82.3	
Dutch {p = 502086, d = 169}	0.0121	0.0101	0,0141	100000	81.3	81.1	81.6	-
Other western {p = 187022, d = 32}	0.0065	0.0041	0,0088	100000	83.5	83.1	84.0	+
Moroccan {p = 128843, d = 29}	0.0092	0.0059	0,0124	100000	89.5	88.1	90.9	+
Antillean {p = 18438, d = 6}	0.0142	0.0039	0,0245	100000	81.3	79.7	83.0	
Surinam {p = 94588, d = 30}	0.0129	0.0084	0,0174	100000	84.0	83.3	84.7	+
Turkish {p = 68886, d = 17}	0.0097	0.0050	0,0143	100000	84.5	83.4	85.7	+
Other non-western {p = 138670, d = 56}	0.0149	0.0107	0,0191	100000	83.8	82.9	84.7	+
Age 40 to 60 (20q40)								
All groups {p = 525628, d = 1235}	0.0494	0.0469	0,0519	98881	42.9	42.7	43.0	
Dutch {p = 259891, d = 744}	0.0581	0.0542	0,0620	98790	42.1	42.0	42.3	-
Other western {p = 84678, d = 170}	0.0434	0.0376	0,0492	99353	44.0	43.6	44.4	+
Moroccan {p = 35502, d = 47}	0.0275	0.0202	0,0349	99087	50.1	48.7	51.5	+
Antillean {p = 7922, d = 21}	0.0545	0.0330	0,0760	98587	42.3	40.9	43.8	
Surinam {p = 61841, d = 145}	0.0477	0.0404	0,0550	98712	44.8	44.2	45.5	+
Turkish {p = 23506, d = 25}	0.0254	0.0172	0,0335	99036	45.1	44.0	46.2	+
Other non-western {p = 52290, d = 83}	0.0340	0.0274	0,0406	98516	44.9	44.0	45.8	+
Age 60 to 80 (20q60)								
All groups {p = 275606, d = 3945}	0.3208	0.3141	0,3276	94077	24.5	24.4	24.7	
Dutch {p = 180803, d = 2888}	0.3437	0.3352	0,3523	93157	24.1	23.9	24.3	-
Other western {p = 41300, d = 495}	0.2776	0.2609	0,2943	95101	25.4	25.1	25.8	+
Moroccan {p = 10561, d = 99}	0.2443	0.2135	0,2750	96383	31.3	29.9	32.7	+
Antillean {p = 3365, d = 41}	0.3147	0.2559	0,3735	93301	24.1	22.7	25.5	
Surinam {p = 21972, d = 266}	0.2901	0.2672	0,3131	94073	26.6	26.0	27.2	+
Turkish {p = 7102, d = 71}	0.2877	0.2495	0,3259	96543	26.0	24.9	27.1	+
Other non-western {p = 10505, d = 85}	0.2331	0.2038	0,2625	95201	26.2	25.3	27.1	+

When comparing males and females, in all ethnic groups and in all age categories males have a higher probability to die compared with females. There is only one exception: in the age group 0 to 40, Antillean females have a higher probability to die compared with Antillean males.

## Conclusions

In this paper we studied life table entries with the aim of getting a better view of differences in mortality between origin groups living in Amsterdam. In this paper as in previous work the life expectancy is mostly higher in non-western compared with western origin groups, however, in this paper females of Dutch origin have a significantly lower expectancy at birth compared with the city average, for Dutch males this is true at age forty and sixty. Separate from the question if this is a healthy migrant, an unhealthy remigration, a registration effect, genetic susceptibility or yet another effect [2], it must be noted that the effect of seemingly lower mortality in non-western groups is not consistent between ethnic group, male citizens of Surinamese origin do significantly wears compared with the ciry average. At a younger age, migrant groups mostly have a relatively high mortality. This is true in terms of the statistical significance of mortality probabilities when larger groups are compared, however, within age, gender and ethnicity sub groups no statistically significant differences between the overall city probability and the subgroups were found. Overall death is rare in young age group. In the different origin group almost all will survive till middle age and the differences in survival are small up to the age of 40. In middle age the number dying rises steeply. Relative mortality differences between origin groups are still large and there are considerable differences in survival between groups. However, the probability to survive the 30 years from 40 to 70 is above 80 % for western and non-western groups alike. As citizens of Dutch origin do not always have a high mortality compared with immigrant groups, the healthy migrant effect is not consistently found in this paper and seems concentrated in the highest age groups. The life table calculation is based on a number of assumptions, and these assumptions might work out different for the different origin groups, particularly in the oldest age group [1, 9, 10]. Part of the difference in older age groups might be caused by migrants having a lower average age within higher age groups, and therefore a lower mortality, compared with the Dutch. A methodological problem causing the healthy migrant effect can therefore not be dismissed in the age groups 80 and above. Males have a higher mortality compared with females in all but one origin group. If one should choose to develop policy or prevention for a particular origin group, such a policy should always consider males first. Lastly, dichotomizing the population in a western non-migrating population and a non-western population of predominantly migrants may be convenient from a research and policy perspective. In this study we find clear differences when such a dichotomy is used. However, within the non-western group the differences in terms of mortality are considerable and a more refined analysis seems required to target resources adequately.

## References

[1] Silcocks PBS, Jenner DA, Reza R (2001). Life expectancy as a summary of mortality in a population: statistical considerations and suitability for use by health authorities. J Epidemiol Community Health.

[2] Uitenbroek DG, Verhoeff AP (2002). Life expectancy and mortality differences between migrant groups living in Amsterdam, The Netherlands. Soc Sci Med.

[3] Khlat M, Darmon N (2003). Is there a Mediterranean migrants mortality paradox in Europe?. Int J Epidemiol.

[4] Razum O (2006). Commentary: of salmon and time travellers--musing on the mystery of migrant mortality. Int J Epidemiol.

[5] Boulogne R, Jougla E, Breem Y, Kunst AE, Rey G (2012). Mortality differences between the foreign-born and locally-born population in France (2004–2007). Soc Sci Med.

[6] Bos V, Kunst AE, Keij-Deerenberg IM, Garssen J, Mackenbach JP (2004). Mortality differences between native Dutch and People of Turkish, Moroccan, Surinamese and Antillean/Aruban Origin. Int J Psychol.

[7] Alders M (2001). Classification of the population with a foreign background in the Netherlands.

[8] Chiang CL, Chiang CL (1968). The life table and its construction. Introduction to Stochastic Processes in Biostatistics.

[9] Eayres D, Williams ES (2004). Evaluation of methodologies for small area life expectancy estimation. J Epidemiol Community Health.

[10] Horiuchi S, Coale AJ (1982). A simple equation for estimating the expectation of life at old ages. Popul Stud (Camb).

